# Pharmacokinetics and Brain Distribution of the Active Components of DA-9805, Saikosaponin A, Paeonol and Imperatorin in Rats

**DOI:** 10.3390/pharmaceutics10030133

**Published:** 2018-08-20

**Authors:** Mi Hye Kwon, Jin Seok Jeong, Jayoung Ryu, Young Woong Cho, Hee Eun Kang

**Affiliations:** 1 College of Pharmacy and Integrated Research Institute of Pharmaceutical Sciences, The Catholic University of Korea, 43 Jibong-ro, Wonmi-gu, Bucheon 14662, Korea; mihye8699@hanmail.net; 2 Research Center, Dong-A ST Co., Ltd., 21 Geumhwa-ro, 105beon-gil, Giheung-gu, Yongin 17073, Korea; treadwheel@donga.co.kr (J.S.J.); jyryu@donga.co.kr (J.R.); herojoe@donga.co.kr (Y.W.C.)

**Keywords:** DA-9805, saikosaponin a, paeonol, imperatorin, pharmacokinetics, brain distribution

## Abstract

DA-9805 is a botanical anti-Parkinson’s drug candidate formulated from ethanol extracts of the root of *Bupleurum falcatum*, the root cortex of *Paeonia suffruticosa*, and the root of *Angelica dahurica*. The pharmacokinetics (PKs) and brain distribution of active/representative ingredients of DA-9805, Saikosaponin a (SSa; 1.1–4.6 mg/kg), Paeonol (PA; 14.8–59.2 mg/kg), and Imperatorin (IMP; 1.4–11.5 mg/kg) were evaluated following the intravenous or oral administration of each pure component and the equivalent dose of DA-9805 in rats. All three components had greater dose-normalized areas under the plasma concentration-time curve (AUC) and slower clearance with higher doses, following intravenous administration. By contrast, dose-proportional AUC values of SSa, PA, and IMP were observed following the oral administration of each pure component (with the exception of IMP at the highest dose) or DA-9805. Compared to oral administration of each pure compound, DA-9805 administration showed an increase in the AUC of SSa (by 96.1–163%) and PA (by 155–164%), possibly due to inhibition of their metabolism by IMP or other component(s) in DA-9805. A delay in the absorption of PA and IMP was observed when they were administered as DA-9805. All three components of DA-9805 showed greater binding values in brain homogenates than in plasma, possibly explaining why the brain-to-plasma ratios were greater than unity following multiple oral administrations of DA-9805. By contrast, their levels in cerebrospinal fluid were negligible. Our results further our understanding of the comprehensive PK characteristics of SSa, PA, and IMP in rats and the comparative PKs between each pure component and DA-9805.

## 1. Introduction

Recently, the potential role of herbal products in the treatment of Parkinson’s disease has emerged [1]. Neuroprotective or neurorestorative agents for the treatment of this disease by either inhibiting primary neurodegenerative events or boosting compensatory and regenerative mechanisms in the brain remain an unmet medical need [2]. DA-9805, a novel botanical neuroprotective anti-Parkinson’s drug candidate, was formulated from ethanol extracts of the mixture (1:1:1, *w*/*w*/*w*) of three herbal drugs, the root of *Bupleurum falcatum* L. (Apiaceae), the root cortex of *Paeonia suffruticosa* Andrews (Paeoniaceae), and the root of *Angelica dahurica* Benth et Hook (Umbelliferae). An investigational new drug application for DA-9805 for a phase II clinical study was recently submitted to the U.S. Food and Drug Administration. The neuroprotective effects of each of the three herbal constituents in DA-9805 have been reported in various in vitro and animal models. Ethanol extracts of the root of *B. falcatum* have been shown to inhibit neuroinflammation in murine microglial cells [3], improve behavioral defects after spinal-cord injury [4], and alleviate stress-induced memory impairment [5]. Extracts from the root cortex of *P. suffruticosa* have been found to have neuroprotective effects in an 1-methyl-4-phenyl-1,2,3,6-tetrahydropyridine (MPTP)-induced Parkinson’s disease model [6]. Extracts from *A. dahurica* reduce apoptotic cell death and improve functional recovery after spinal cord injury [7].

**Figure 1:**
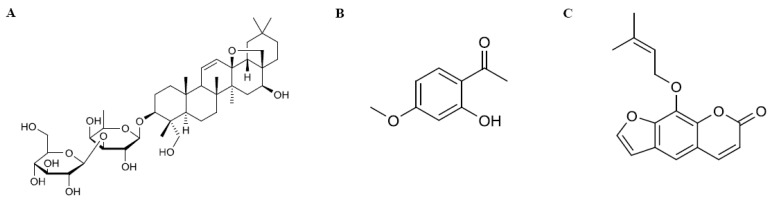
Chemical structures of three active/representative components in DA-9805: (**A**) saikosaponin a (SSa); (**B**) paeonol (PA); (**C**) imperatorin (IMP).

Saikosaponin a (SSa, [3β,4α,16β]-13,28-epoxy-16,23-dihydroxyolean-11-en-3-yl-6-deoxy-3-*O*-β-d-glucopyranosyl-β-d-galactopyranoside; Figure 1A), originated from the root of *B. falcatum*, is a representative component of DA-9805, for which it is used as a quality control (QC) ingredient (at >0.297%). The quantity of SSa is listed in the Chinese Pharmacopeia for QC of a traditional Chinese medicine (TCM) prescribed with Radix Bupleuri [8]. Its pharmacological activity and structure are similar to those of steroids [9], and its antiepileptic [10] and neuroprotective effects [11] have recently been reported. However, its toxicity, including an ability to cause hemolysis and liver damage, has also been reported [12]. The pharmacokinetics (PKs) of SSa following intravenous administration of 15 mg/kg [8] and oral administration of the extract of the dried roots of *B. chinense* DC (equivalent to ~40 mg/kg SSa) [13] have been reported.

Paeonol (PA; 1-[2-hydroxy-4-methoxyphenyl]ethanone; Figure 1B), a marker component of the root cortex of *P. suffruticosa* [14], is the most abundant active compound in DA-9805 (at >2.802%). It has various pharmacological activities [15,16,17] and neuroprotective effects in various murine models [18,19,20]. Its PKs and/or tissue distribution have been reported after intravenous (2.5–10 mg/kg) [21] and oral administration (35–140 mg/kg) [14,22], as well as after oral administration of herbal products containing PA or TCM prescriptions (equivalent to 17.5–100 mg/kg PA) [23,24].

Imperatorin (IMP, 9-[3-methylbut-2-enoxy]-7-furo[3,2-g]chromenone; Figure 1C), an active ingredient in the root of *A. dahurica*, is another representative component of DA-9805 (at >0.512%). It has anti-inflammatory effects [25,26] and other favorable effects in the central nervous system (CNS) including neuroprotective, anticonvulsant, anxiolytic, and procognitive activities [27,28,29]. Its PKs following intravenous and oral administration (6.25–25 mg/kg or 80 mg/kg) in rats have been reported [30,31], as has its brain distribution after oral administration of 20 mg/kg of IMP [32] or ~26 mg/kg in an extract [33].

The efficacy and safety of herbal medicines remain major issues of concern. Up to now, a widely accepted approach for evaluating in vivo efficacy of an herbal medicine is that the efficacy of mixed herbal preparation can be correlated with pharmacokinetic behavior of one or several known active ingredients [34]. PKs studies of these three active/representative ingredients of DA-9805 are essential for botanical drug development to evaluate not only the efficacy of the drug but also its potential drug interactions and toxicity. Despite the promising neuroprotective activities of DA-9805 as an anti-Parkinson’s agent, the PKs of its active/representative ingredients at relatively low doses have not been characterized comprehensively. Moreover, PKs of each active ingredient following DA-9805 administration may be altered by the multiple components in DA-9805.

In this study, the PKs of pure SSa, PA, and IMP and their dose dependency were evaluated following the intravenous and oral administration of each pure component. The PKs of each marker compound following the oral administration of DA-9805 were also compared to those following the respective equivalent dose of the pure marker components. Moreover, the distribution of the three active components in the brain, which is the site of action, was evaluated following multiple oral administrations of DA-9805.

## 2. Materials and Methods

### 2.1. Materials and Reagents

DA-9805 (Lot No. PD15702; containing 0.456% SSa, 5.919% PA, and 0.576% IMP, as determined by HPLC-UV) was obtained from Dong-A ST (Yongin, Korea). SSa (98.32% purity) and IMP (99.17% purity) were products from Letopharm, Ltd. (Shanghai, China), and PA (99.8% purity) was obtained from Sigma-Aldrich (St. Louis, MO, USA). Internal standard (IS) for SSa, PA, and IMP (dexamethasone, bumetanide, indomethacin, respectively), hydroxypropyl-methylcellulose (HPMC), dimethylsulfoxide (DMSO), Tween^®^ 80, and Solutol^®^ HS 15 were purchased from Sigma-Aldrich (St. Louis, MO, USA). Other chemicals were of reagent or HPLC grade.

### 2.2. Animals

Animal-study protocols were approved by the Department of Laboratory Animals, Institutional Animal Care and Use Committee on the Sungsim Campus of the Catholic University of Korea (Approval No. 2015-011; Bucheon, Korea). Male Sprague-Dawley rats, 7–8 weeks old and weighing 195–300 g, were purchased from Young Bio (Sungnam, Korea). The procedures used for the housing and handling of rats were similar to those previously described [35].

### 2.3. Intravenous Drug Administration

For intravenous administration, the carotid artery and jugular vein were cannulated using previously reported procedures [35]. Thereafter, each pure active component of DA-9805 was infused for 1 min via the jugular vein: SSa (dissolved in 2:1:7 [*v*/*v*/*v*] DMSO:Tween 80:distilled water (DW)) at doses of 2.3 and 4.6 mg (2 mL)/kg (*n* = 5 and 4, respectively); PA (dissolved in 2:2:6 [*v*/*v*/*v*] DMSO:Tween 80:20% Solutol in DW) at doses of 14.8, 29.6, and 59.2 mg (2 mL)/kg (*n* = 5, 5, and 6, respectively); and IMP (dissolved in 2:2:6 [*v*/*v*/*v*] DMSO:Tween 80: DW) at doses of 2.9, 5.8, and 11.5 mg (2 mL)/kg (*n* = 5, 6 and 5, respectively). A blood sample of ~200 µL was collected via the carotid artery at time 0 (prior to dosing), 1 (at the end of the infusion), and 5, 15, 30, 60, 90, 120, 180, 240, 360, and 480 min after the start of infusion. After centrifugation (13,000× *g* for 2 min), 100 µL aliquots of plasma were collected and stored at −20 °C until liquid chromatography-tandem mass spectrometry (LC-MS/MS) analyses. The preparation and handling of 24 h urine samples (Ae_0–24h_) and gastrointestinal tract (GI) samples (including their contents at 24 h and feces during 0–24 h) at 24 h (GI_24h_) were similar to a previously reported method [35].

### 2.4. Oral Drug Administration

DA-9805 (dissolved in 2:7.75, 2:7.5, and 2:7 [*v*/*v*] DMSO:DW with 2% HPMC, respectively) at doses of 250, 500, and 1000 mg (10 mL)/kg was administered orally using a gastric gavage tube (*n* = 5 each). Equivalent doses of each pure SSa, PA, and IMP (dissolved in 2:7.75:0.25, 2:7.5:0.5, and 2:7:1 [*v*/*v*/*v*] DMSO:DW with 2% HPMC:ethanol with dose elevation) were also administered orally (*n* = 4 or 5 each). Doses of 1.1, 2.3, and 4.6 mg (10 mL)/kg pure SSa; 14.8, 29.6, and 59.2 mg (10 mL)/kg pure PA; and 1.4, 2.9, and 5.8 mg (10 mL)/kg pure IMP were orally administered. A blood sample of ~200 µL was collected via the carotid artery at 0, 5, 15, 30, 45, 60, 90, 120, 180, 240, 360, 480, and 600 min after oral administration. Other procedures for the oral study were similar to those used for the intravenous study.

### 2.5. Brain Distribution of Saikosaponin A(SSa), Paeonol (PA), and Imperatorin (IMP) Following Multiple Oral Doses of DA-9805

DA-9805 (dissolved in 2:7 [*v*/*v*] DMSO:DW with 2% HPMC and diluted 2-fold with DW to a final concentration of 250 mg/5 mL) at a dose of 250 mg (5 mL)/kg/day was orally administered for 8 days. At 2 and 6 h after the final oral dose, the rats were euthanized (*n* = 3 at each time point) by carbon-dioxide asphyxiation, and cerebrospinal fluid (CSF) was collected from each rat. Blood samples were collected via cardiac puncture. Next, the mixed brain (whole of left hemisphere), frontal cortex, striatum, hippocampus, and cerebellum (from the right hemisphere) were excised and blotted on tissue paper. Each tissue sample was homogenized (Minilys^®^; Bertin Technologies, Montigny le Bretonneux, France) with 3 volumes (3 mL/g tissue) of methanol and centrifuged at 9000 g for 10 min. Two 100 µL aliquots of the supernatant and plasma samples were collected and stored at −70 °C until subsequent LC-MS/MS analyses.

### 2.6. Measurement of the Protein Binding of SSa, PA, and IMP in Rat Plasma and Brain Homogenates

The protein-binding abilities of SSa, PA, and IMP in fresh rat plasma and brain homogenates were measured using a single-use rapid equilibrium dialysis device (molecular weight cutoff of 8 kDa; Thermo Scientific, Rockford, IL, USA) in accordance with the manufacturer’s instructions. For binding studies with rat plasma, SSa, PA, and IMP were spiked into each 1200 µL blank plasma sample (*n* = 6) to produce a final concentration of 0.5, 2, and 0.5 μg/mL, respectively. A triplicate of the above samples (each containing 300 µL) was placed into each sample chamber to minimize potential errors during sample processing, and was dialyzed against 500 µL isotonic phosphate buffer (pH 7.4) in each buffer chamber. After 4 h incubation in a water bath shaker (37 °C, 50 oscillations/min), the plasma and buffer samples were collected, and each 100 µL sample was used for LC-MS/MS analyses of SSa, PA, and IMP. For the binding study with rat brain (hippocampus, striatum, mixed brain) homogenates, each brain tissue sample was homogenized (Minilys^®^; Bertin Technologies, Montigny le Bretonneux, France) with 3 volumes (3 mL/g tissue) of isotonic phosphate buffer (pH 7.4). SSa, PA, and IMP were spiked into each blank brain homogenate to produce a final concentration of 0.2, 1, and 0.2 μg/mL, respectively. One (for the hippocampus and striatum) and a duplicate (for the mixed brain) of the above homogenate samples (each 200 µL) were transferred into each sample chamber and dialyzed against 350 µL isotonic phosphate buffer (pH 7.4) in each buffer chamber. Other procedures were the same as those used in the plasma protein-binding study. The unbound fraction (*f*_u_) in plasma was calculated as follows:

*f*_u_ = 1 − (*C*_p_ − *C*_b_)/*C*_p_, (1)

where *C*_p_ is the drug concentration in the protein-containing compartment and *C*_b_ is the drug concentration in the buffer compartment. Because the brain homogenates were diluted during the homogenization process, it was necessary to correct the *f*_u_ value measured in a diluted sample (*f*_u measured_) to generate the undiluted *f*_u_ value (*f*_u brain_) using the following equation (Kalvass and Maurer, 2002):

(2) $$f_{u\;brain}=\;\frac{1/D}{\left\{\left(1/f_{u\;measured}\right)-1\right\}+1/D},$$

where *D* is the dilution factor (=4) of the brain homogenates.

### 2.7. LC-MS/MS Analyses of SSa, PA, and IMP

The LC-MS/MS system comprised the Agilent 1260 LC System and the Agilent 6460 Triple Quadrupole Tandem Mass Spectrometer (Agilent, Waldbronn, Germany). Instrument control and data acquisition were performed using the Agilent MassHunter Workstation software (Version B. 04. 01). The concentrations of SSa, PA, and IMP in the samples were determined according to the LC-MS/MS method recently published from our laboratory [36]. The calibration curves were linear in a concentration range of 0.5–1000 ng/mL for SSa, 20–10,000 ng/mL for PA, and 0.2–1000 ng/mL for IMP. The mean intra- and interday coefficients of variation of the analyses on 5 consecutive days were below 15.0%, and the assay accuracies ranged from 95.8 to 113%.

### 2.8. Pharmacokinetic Analyses

The total area under the plasma concentration–time curve from time zero to infinity (AUC_0–inf_) or up to the last measured time (*t*) in plasma (AUC_0–t_) was calculated using the trapezoidal rule-extrapolation method [37]. Calculation of the following PK parameters was performed using non-compartmental analyses (WinNonlin^®^; Pharsight Corporation, Mountain View, CA, USA): the time-averaged total body, renal, and nonrenal clearances (CL, CL_R_, and CL_NR_, respectively), terminal half-life, mean residence time (MRT), and apparent volume of distribution at steady state (*V*_ss_) [38]. The maximum plasma concentration (*C*_max_) and time to reach *C*_max_ (*T*_max_) were directly read from the experimental data. For comparison, the extent of absolute oral bioavailability (F) was calculated by dividing the AUC value after oral administration by the AUC value after intravenous administration of equivalent doses of the pure component.

### 2.9. Statistical Analyses

The Statistical Package for Social Sciences program (SPSS Inc., Chicago, IL, USA) was used for statistical analyses. To compare two means of unpaired data, the t-test was used. To compare 3 means of unpaired data, the data were compared using one-way analysis of variance (ANOVA) and the post hoc Tukey test (for a homogenous subset), or Dunnett’s T3 multiple-comparison test (for a non-homogenous subset). For data that were not normally distributed, nonparametric analyses (Kruskal-Wallis test) were used. A *p* value < 0.05 was considered statistically significant. All of the data are expressed as the means ± standard deviations (SD), except the median (range) for *T*_max_.

## 3. Results

### 3.1. PKs of SSa, PA, and IMP after Intravenous Administration of Each Pure Compound

**Figure 2:**
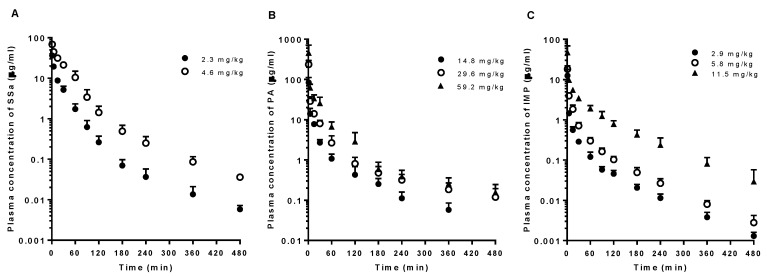
Mean arterial plasma concentration-time profiles of (**A**) SSa, (**B**) PA, and (**C**) IMP following 1-min intravenous administration of each pure compound at various doses (2.3 (*n* = 5) and 4.6 (*n* = 4) mg/kg of SSa; 14.8 (*n* = 5), 29.6 (*n* = 5), and 59.2 (*n* = 6) mg/kg of PA; and 2.9 (*n* = 5), 5.8 (*n* = 6), and 11.5 (*n* = 5) mg/kg of IMP) to rats. Vertical bars represent SD.

The mean arterial plasma concentration-time profiles and relevant PK parameters of SSa, PA, and IMP following intravenous administration of each pure compound are shown in Figure 2 and Table 1, respectively. The dose-normalized AUC values of SSa, PA, and IMP following intravenous administration tended to increase with dose elevation because of slower CL (particularly CL_NR_) of the drugs in the higher dose group(s). A significantly greater (by 77.9%) dose-normalized AUC value of SSa following intravenous administration of 4.6 mg/kg SSa was observed with significantly slower CL (by 43.2%), CL_R_ (by 81.2%), and CL_NR_ (by 40.3%) than those of the 2.3 mg/kg dose group. A significantly smaller *V*_ss_ (by 19.0%), longer MRT (by 42.9%), and smaller Ae_0–24h_ (by 68.0%) of SSa were observed in the higher dose group. PA showed significantly greater (by 38.0%) dose-normalized AUC with slower CL (by 27.0%) and CL_NR_ (by 27.5%) in the highest intravenous dose (59.2 mg/kg) group than in the lowest dose (14.8 mg/kg) group. IMP also showed significantly greater (by 124% and 98.5%) dose-normalized AUC following the highest intravenous dose (11.5 mg/kg) with slower CL (by 56.4% and 50.5%) and CL_NR_ (by 56.4% and 50.5%) than the lower dose (2.9 and 5.8 mg/kg) groups. A significantly longer MRT (by 96.6% and 60.2%) was observed in the highest dose (11.5 mg/kg) IMP group than in the lower dose (2.9 and 5.8 mg/kg) groups. The 24 h urinary excretion of IMP was almost negligible (<0.1% of the intravenous dose).

### 3.2. PKs of SSa after the Oral Administration of Pure SSa and DA-9805

**Figure 3:**
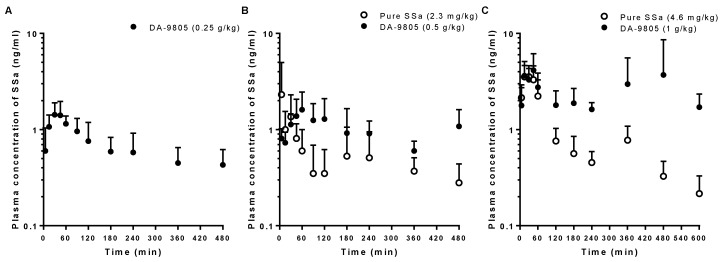
Mean arterial plasma concentration–time profiles of SSa following oral administration of DA-9805 (closed circle) at doses of 0.25 g/kg ((**A**); 1.1 mg/kg as SSa, *n* = 5), 0.5 g/kg ((**B**); 2.3 mg/kg as SSa, *n* = 5), and 1 g/kg ((**C**); 4.6 mg/kg as SSa, *n* = 5) and pure SSa (open circle) at doses of 2.3 mg/kg ((**B**); *n* = 4) and 4.6 mg/kg ((**C**); *n* = 5) to rats. Vertical bars represent SD.

The mean arterial plasma concentration-time profiles of SSa after oral administration in rats of pure SSa at doses of 2.3 and 4.6 mg/kg and DA-9805 at doses of 0.25, 0.5, and 1 g/kg (equivalent to 1.1, 2.3, and 4.6 mg/kg, respectively, as SSa) are shown in Figure 3. The relevant PK parameters are also listed in Table 2. Most of the plasma concentrations of SSa following oral administration of pure SSa at the lowest dose (1.1 mg/kg) were less than the lower limit of quantification (LLOQ; 0.5 ng/mL); thus, the data were excluded for PK analyses. The AUC and *C*_max_ values of SSa were dose-proportional following oral administration of pure SSa and DA-9805. Interestingly, the AUC_0–8h_ values of SSa following oral administration of DA-9805 were significantly greater (by 96.1% and 163%) than those following oral administration of each equivalent dose of pure SSa, whereas the *C*_max_ values were comparable between pure SSa and DA-9805. Therefore, the F values were greater (by 96.2% and 163%) following DA-9805 administration than after pure SSa administration. The F values of SSa following oral administration were extremely low (<0.1% of oral dose). Interestingly, the recovery from the GI tract (GI_24 h_ values) was significantly higher (24.5- and 3.1-fold) in rats with DA-9805 administration than in rats with pure SSa administration.

### 3.3. PKs of PA after the Oral Administration of Pure PA and DA-9805

**Figure 4:**
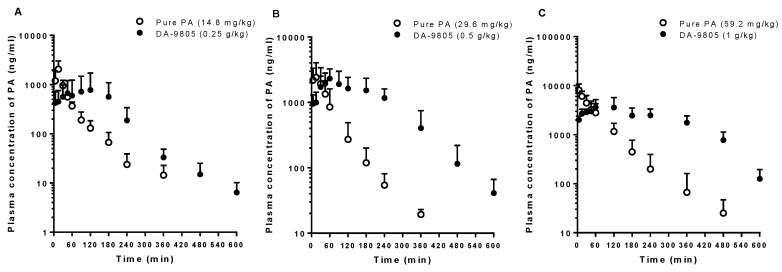
Mean arterial plasma concentration–time profiles of PA following oral administration of DA-9805 (closed circle) at doses of 0.25 g/kg (**A**; 14.8 mg/kg as PA, *n* = 5), 0.5 g/kg (**B**; 29.6 mg/kg as PA, *n* = 5), and 1 g/kg (**C**; 59.2 mg/kg as PA, *n* = 5) mg/kg and equivalent doses of pure PA (open circle; 14.8 mg/kg (**A**; *n* = 4), 29.6 mg/kg (**B**; *n* = 5), and 59.2 mg/kg (**C**; *n* = 4)) to rats. Vertical bars represent SD.

The mean arterial plasma concentration-time profiles of PA in rats after oral administration of pure PA at doses of 14.8, 29.6, and 59.2 mg/kg and equivalent doses of DA-9805 are shown in Figure 4. The relevant PK parameters are listed in Table 3. The AUC and *C*_max_ values of PA were proportional to PA doses following oral administration of pure PA and DA-9805. The AUC values of PA following oral administration of 0.5 and 1 g/kg of DA-9805 were significantly greater (by 164% and 155%) than those following the oral administration of each equivalent dose of pure PA, whereas the *C*_max_ values were comparable between pure PA and DA-9805 or even less than those in the pure PA group. Again, greater F values of PA were observed following DA-9805 administration than after pure PA administration (28.7–41.7% vs. 12.2–16.4%). Following the administration of DA-9805, slow absorption of PA supported by delayed *T*_max_ was observed compared to pure PA administration.

### 3.4. PKs of IMP after the Oral Administration of Pure IMP and DA-9805

**Figure 5:**
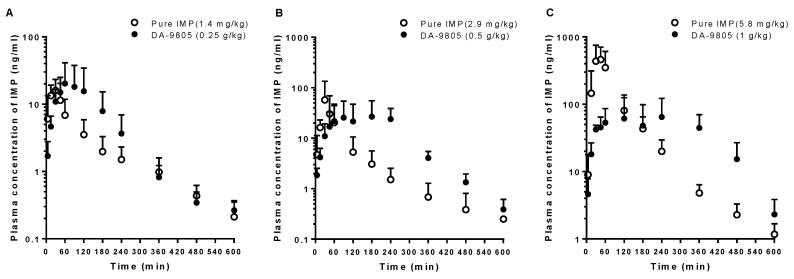
Mean arterial plasma concentration–time profiles of IMP following oral administration of DA-9805 (closed circle) at doses of 0.25 g/kg (**A**; 1.4 mg/kg as IMP, *n* = 5), 0.5 g/kg (**B**; 2.9 mg/kg as IMP, *n* = 5), and 1 g/kg (**C**; 5.8 mg/kg as IMP, *n* = 5) mg/kg and equivalent doses of pure IMP (open circle; 1.4 mg/kg (**A**; *n* = 6), 2.9 (**B**; *n* = 6), and 5.8 mg/kg (**C**; *n* = 5)) to rats. Vertical bars represent SD.

The mean arterial plasma concentration-time profiles of IMP after oral administration in rats of pure IMP at doses of 1.4, 2.9, and 5.8 mg/kg and equivalent doses of DA-9805 are shown in Figure 5. The relevant PK parameters are listed in Table 4. Following the oral administration of pure IMP, the dose-normalized AUC and *C*_max_ values of IMP at the highest level (5.8 mg/kg) were significantly greater (by 471% and 513%) than those in the lower dose groups (1.4 and 2.9 mg/kg). By contrast, DA-9805 showed dose-proportional AUC and *C*_max_ values of IMP in the equivalent oral dose range (0.25–1 g/kg; 1.4–5.8 mg/kg as IMP). Comparable AUC values of IMP were observed between the DA-9805 and pure IMP administration groups. Significantly delayed *T*_max_ values and/or lowered *C*_max_ values were observed following DA-9805 administration compared to those after each equivalent dose of pure IMP administration.

### 3.5. Protein Binding of SSa, PA, and IMP in Rat Plasma and Brain-Tissue Homogenates

**Figure 6:**
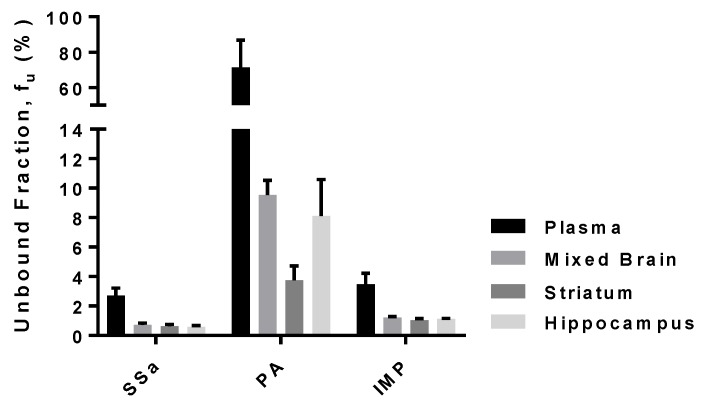
Mean unbound fraction (*f*_u_, %) of SSa, PA, and IMP in rat plasma and brain (mixed brain, striatum, hippocampus) homogenates (*n* = 6 each). Vertical bars represent SD.

The unbound fraction of SSa, PA, and IMP in rat plasma and brain-tissue homogenates are shown in Figure 6. The plasma protein-binding values of SSa, PA, and IMP were 97.3 ± 0.516% (free fraction of 2.7%), 28.5 ± 15.4% (free fraction of 71.5%), and 96.5 ± 0.744% (free fraction of 3.5%), respectively. Nonspecific binding of SSa, PA, and IMP to the equilibrium dialysis devices was negligible because their recovery after incubation was almost complete (>94.7%). The binding values of all three compounds were higher in brain homogenates than in plasma. The binding values of SSa in each brain tissue (striatum, hippocampus, and mixed brain) homogenate were 99.4 ± 0.138%, 99.4 ± 0.099%, and 99.3 ± 0.124% (free fraction of 0.618, 0.582, and 0.719%), respectively. The binding values of PA in the striatum, hippocampus, and mixed brain homogenates were 96.3 ± 0.957%, 91.9 ± 2.49%, and 90.5 ± 0.996% (free fraction of 3.75%, 8.10%, and 9.53%), respectively; and the corresponding IMP values were 99.0 ± 0.10%, 98.9 ± 0.037%, and 98.8 ± 0.068% (free fraction of 1.04%, 1.12%, and 1.22%), respectively.

### 3.6. Brain and CSF Distribution of SSa, PA, and IMP after the Oral Administration of DA-9805

**Figure 7:**
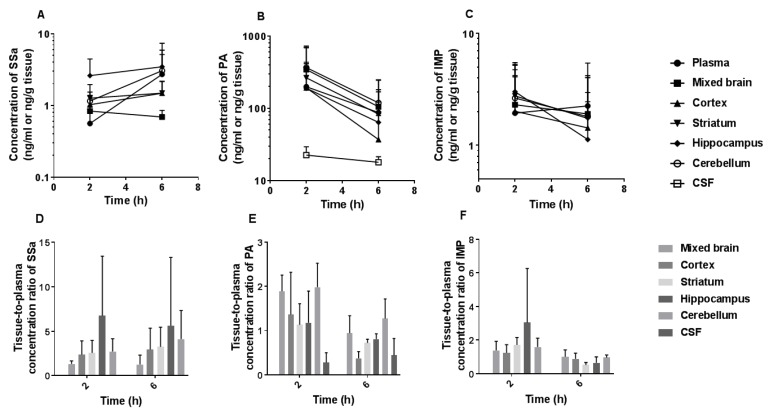
Mean plasma and brain regional concentration–time profiles of (**A**) SSa, (**B**) PA, and (**C**) IMP and their tissue-to-plasma (T/P) concentration ratios (**D**, **E**, and **F** for SSa, PA, and IMP, respectively) following multiple oral administration of DA-9805 (250 mg/kg/day for 8 days) at 2 h (*n* = 5) and 6 h (*n* = 4) after the final dose. Vertical bars represent SD.

The mean plasma, brain regional (frontal cortex, striatum, hippocampus, and cerebellum), and CSF concentration–time profiles and tissue-to-plasma (T/P) ratios of SSa, PA, and IMP after multiple oral administration of DA-9805 (250 mg/kg/day for 8 days) are shown in Figure 7. The T/P ratios of SSa in each brain region were greater than unity (1.26–6.73) at 2 and 6 h, whereas the concentrations of SSa in CSF were less than the LLOQ. For PA, the T/P ratios in each brain region were slightly greater than unity (1.13–1.98) at 2 h after the final DA-9805 oral dose, whereas the values, with the exception of those for the cerebellum, became smaller than unity at 6 h. The PA levels in the CSF were notably lower than those in the brain tissues and plasma. The T/P ratios of IMP in each brain region were greater than unity (1.24–3.04) at 2 h after the final dose, whereas the values became smaller than unity at 6 h. The concentrations of IMP in CSF were below the LLOQ.

## 4. Discussion

The effective oral dose of DA-9805 is 10–30 mg/kg/day in mice, and the planned dose regimen in the clinical study would be 30 mg or 60 mg three times a day (internal reports). However, the PKs of DA-9805 at this effective dose range could not be investigated due to the extremely low concentrations (<1%) of the two representative ingredients, SSa and IMP, in DA-9805. The doses of SSa (1.1–4.6 mg/kg), PA (14.8–59.2 mg/kg), and IMP (1.4–11.5 mg/kg) used in this study were determined based on the maximal tolerated dose of DA-9805, their concentrations in DA-9805, and assay sensitivity of each substance. Based on the relationship between the dose and PK parameters, the PKs of each component of DA-9805 in the effective dose range could be estimated.

The plasma concentrations of SSa after its intravenous administration at doses of 2.3 and 4.6 mg/kg decreased in a polyexponential fashion (Figure 2A). Based on the slower CL (primarily CL_NR_) of SSa and greater dose-normalized AUC in the higher dose group, SSa was eliminated via a saturable process. The CL values of SSa were relatively slow compared to those of PA and IMP. Although the contribution of CL_R_ to CL was small (2.22–6.94%), renal excretion of SSa seemed to also be saturated in the higher dose group based on the slowed CL_R_. The CL_R_ of SSa as the free (unbound to plasma proteins) fraction (CL_R,fu_) was estimated based on the CL_R_ (Table 1) and free fraction of SSa in plasma. The estimated value was 2.23–11.9 mL/min/kg, similar to the reported glomerular filtration rate in rats at 5.24 mL/min/kg [39]. The *V*_ss_ values of SSa (102–126 mL/kg) were relatively small considering that the total body water in rats is 668 mL/kg [39]; hence, the distribution of SSa in tissues might be restricted due to its hydrophilicity, large molecular weight, and high binding affinity to plasma protein. 

Interestingly, SSa showed linear PKs following the oral administration of pure SSa and DA-9805 based on the dose-proportional AUC and *C*_max_ values. This is because of the linear elimination of SSa at much lower plasma concentrations of SSa following oral administration in contrast to the saturation of SSa elimination following intravenous administration. The dose-normalized AUC of SSa following oral administration of 4.6 mg/kg of pure SSa is also similar to the dose-normalized value in previous reports with a much higher dose in TCM (~42 mg/kg as SSa) [13]. Note that the F values of SSa following oral administration were extremely low (<0.1%). Slow SSa CL (Table 1) suggested that its first-pass metabolism is not considerable. Therefore, the extremely low F value of SSa was most likely due to incomplete absorption. The low permeability of SSa because of its large molecular weight (>700 Da) and hydrophilicity may contribute to incomplete absorption. Considering the extremely low F values of SSa, its recovery from the GI tract (GI_24 h_ values, Table 2) seems incomplete due to possible decomposition in the GI tract. SSa, which includes an unstable allyl oxide linkage, could be converted into diene saponin by gastric juice [40]. In addition, both the SSa and diene-transformed saikosaponins could be stripped of their sugar moieties by intestinal flora [40,41]. Therefore, the decomposition of SSa in the GI tract could also be one of the possible reasons for the incomplete absorption.

The plasma concentrations of PA after its intravenous administration at dose ranges of 14.8–59.2 mg/kg also decreased in a polyexponential fashion (Figure 2B). PA showed slower CL (primarily CL_NR_) and greater dose-normalized AUC values at the highest dose than at the lowest dose. This indicates that the elimination of PA is saturated at the highest dose. Intravenous PA over the lower dose range of 2.5–10 mg/kg showed linear PKs supported by constant CL (49.3–53.2 mL/min/kg) and dose-proportional AUC values [21]. The CL values of PA (21.4–29.3 mL/min/kg) obtained in this study (dose range of 14.8–59.2 mg/kg), which are slower than those from a previous report, again suggest the saturation of elimination at a higher dose range in this study. The CL_R_ of PA as a free (unbound to plasma proteins) fraction (CL_R,fu_) was estimated based on the CL_R_ (Table 1) and free fraction of PA in plasma. Thus, the CL_R,fu_ values estimated were 0.324–0.821 mL/min/kg, slower than the reported glomerular filtration rate in rats (5.24 mL/min/kg) [39]. The above data indicate that PA was primarily reabsorbed from the renal tubules in rats. Because the contribution of CL_R_ of PA to CL was negligible (0.941–2.55%), nonrenal elimination was concluded to be the major elimination route of PA. The *V*_ss_ values of PA (1100–1290 mL/kg) are greater than the reported total body water in rats (668 mL/kg) [39]. Unlike following intravenous administration, PA showed linear PKs following the oral administration of pure PA and DA-9805 at a dose range of 14.8–59.2 mg/kg as PA. The linear oral PKs of PA have been reported with a pure PA dose range of 35 to 140 mg/kg in rats [22]. 

The plasma concentrations of IMP after its intravenous administration at a dose range of 2.9–11.5 mg/kg also decreased in a polyexponential fashion (Figure 2C). Slowed CL (primarily CL_NR_) and increased dose-normalized AUC values of IMP in the highest dose group suggested the saturation of its elimination at the highest dose. The almost negligible recovery from urine (Ae_0–24h_) and CL_R_ of IMP (Table 1) indicates that the major elimination route of IMP is a nonrenal process. IMP showed the greatest *V*_ss_ values among the three components of DA-9805, which suggests that it has the highest affinity to tissue(s). Following oral administration of pure IMP, the significantly greater dose-normalized AUC and *C*_max_ values of IMP at the highest level (5.8 mg/kg) than those in the lower-dose groups (1.4 and 2.9 mg/kg) could be a result of the saturation of first-pass metabolism of IMP at the highest dose.

When the PKs of each component following oral administration of DA-9805 were compared to the administration of each equivalent dose of pure component, several significant and interesting differences were found. First, following the oral administration of DA-9805, significantly higher plasma concentrations, and hence, greater AUC and F values of SSa and PA, were observed than those following oral administration of each equivalent dose of pure SSa and PA. This suggests the significant influence of DA-9805 component(s) on the PKs of SSa and PA. Among the components of DA-9805, IMP shows inhibitory effects on CYP1A2 and 2B6 in both human and rat liver microsomes [42]. It also has weaker inhibitory effects on other major human (2C19, 2C9, 2D6, and 3A4) and rat (2D2 and 3A1/2) CYP isozymes. O-demethylation of PA is the major in vivo metabolic pathway in rats [22], and the major isoform responsible for O-demethylation of PA in human liver microsomes is CYP1A2 [43]. Therefore, it is likely that IMP in DA-9805 inhibits the hepatic metabolism of PA via CYP1A2 and results in greater AUC values of PA following DA-9805 administration than after pure PA administration. SSa is also mainly metabolized in a Phase I manner [44]. Although the enzymes responsible for this Phase I metabolism of SSa have not been reported, the inhibition of SSa metabolism by IMP or other component(s) in DA-9805 could result in increased AUCs following DA-9805 administration.

Second, significantly more SSa was recovered from the GI tract at 24 h (including feces collected for 24 h) after the oral dose (GI_24 h_ values in Table 2) when DA-9805 was administered compared to pure SSa administration. This may not be due to decreased absorption of SSa when DA-9805 was administered compared to when pure SSa was administered because even greater AUC and F values of SSa were observed in rats with DA-9805 administration. Possibly, other saikosaponins and sugar-containing glycosides in DA-9805 could competitively prevent the decomposition of SSa in the GI lumen such as the hydrolysis of sugar moieties by intestinal flora. 

Finally, the absorption of PA and IMP seemed to be delayed following oral administration of DA-9805 supported by their delayed *T*_max_ values compared to the administration of each pure component. Multiple components in DA-9805 might influence the absorption rate of each component. One of the possible reasons for the slowed absorption of PA and IMP could be slowed GI motility following DA-9805 administration because both Radix Paeoniae [45,46] and Radix Bupleuri [47] have antispasmodic effects on the GI tract. Because of the delayed absorption of IMP following DA-9805 administration, the *C*_max_ value of IMP is lowered and might not reach the level to cause saturation of its first-pass metabolism. This explains linear PKs of IMP up to the highest oral dose, when DA-9805 was administered. By contrast, following the highest oral dose (5.8 mg/kg) of pure IMP, possible saturation of its first-pass metabolism may result in greater dose-normalized AUCs than the lower dose groups. 

Enhanced exposure of SSa and PA and delayed absorption of PA and IMP when administered as DA-9805 resulted in favorable PK profiles (i.e., elevated and sustained plasma concentrations of the three active components). The different PKs of the active components when administered as DA-9805 suggest possibly superior efficacy of DA-9805 over each pure component or their simple mixture.

The binding values of SSa and IMP to plasma protein were considerable (97.3% and 96.5%, respectively), whereas PA has relatively low binding values. However, the binding values of SSa, PA, and IMP were all considerable (>90.5%) in mixed brain, striatum, and hippocampus homogenates. Note that all three components of DA-9805 showed greater binding values (smaller free fraction) in brain homogenates than in plasma, possibly enabling their higher concentrations in the brain than in plasma. This result is consistent with the tissue-to-plasma ratios of the three components being greater than unity in most brain tissues at 2 h following multiple oral administrations of DA-9805. PA and IMP pass through the blood–brain barrier [14,32]. The concentrations of IMP in the striatum and hippocampus are higher than those in other regions [32]; our results are consistent with those findings. Notably, a brain-to-plasma ratio greater than unity of SSa suggests the possibility that SSa passes through the blood–brain barrier, despite its high polarity and large molecular weight, in line with previous studies reporting pharmacological activities of SSa in CNS [10,11]. By contrast, extremely low concentrations of the three components in the CSF suggest that the distribution of the three components in the CSF is negligible.

## 5. Conclusions

Following the intravenous administration of each pure component of DA-9805 (SSa, PA, and IMP), all three components showed dose-dependent PKs because of the saturation of elimination at a higher dose. By contrast, the dose-proportional AUC values of SSa, PA, and IMP were observed following oral administration of each pure component (except IMP at the highest dose) or DA-9805. Compared to the oral administration of each pure compound, oral administration as DA-9805 showed an increase in the AUC of SSa and PA, possibly due to inhibition of their metabolism by IMP or other component(s) in DA-9805. A delay in the absorption of PA and IMP was also observed when they were administered as DA-9805. The binding values of SSa and IMP to plasma protein or brain homogenates were considerable, whereas PA had relatively low binding values in plasma. All three components showed greater binding values in brain homogenates than in plasma, possibly explaining the observed brain-to-plasma ratios being greater than unity following multiple oral administrations of DA-9805 in contrast to their negligible levels in the CSF. This study furthers our understanding of the comprehensive PKs of SSa, PA, and IMP in rats and comparative oral PKs between each pure component and DA-9805.

## Figures and Tables

**Table 1:** Pharmacokinetic parameters of Saikosaponin a (SSa), paeonol (PA), and imperatorin (IMP) after intravenous administration of each drug to rats (mean ± standard deviations (SD)).

Parameters	SSa		PA			IMP		
	2.3 mg/kg (*n* = 5)	4.6 mg/kg (*n* = 4)	14.8 mg/kg (*n* = 5)	29.6 mg/kg (*n* = 5)	59.2 mg/kg (*n* = 6)	2.9 mg/kg (*n* = 5)	5.8 mg/kg (*n* = 6)	11.5 mg/kg (*n* = 5)
Body weight (g)	290 ± 7.91	288 ± 8.66	262 ± 7.58	263 ± 12.5	258 ± 7.58	274 ± 10.7	280 ± 10.5	289 ± 11.9
AUC_0–inf_ (μg∙min/mL) ^a^	517 ± 64.3	1840 ± 349 ^b^	511 ± 65.3	1250 ± 250	2820 ± 400 ^f^	55.9 ± 12.4	126 ± 23.9	496 ± 66.5 ^h^
CL (mL/min/kg)	4.51 ± 0.583	2.56 ± 0.418 ^c^	29.3 ± 3.60	24.4 ± 4.03	21.4 ± 3.29 ^g^	53.9 ± 11.9	47.5 ± 9.85	23.5 ± 3.07 ^i^
CL_R_ (μL/min/kg)	321 ± 177	60.2 ± 34.2 ^d^	587 ± 535	232 ± 110	556 ± 382	39.4 ± 9.33	42.5 ± 27.7	13.8 ± 4.35
CL_NR_ (mL/min/kg)	4.19 ± 0.492	2.50 ± 0.387 ^c^	28.7 ± 3.75	24.2 ± 3.98	20.8 ± 3.16 ^g^	53.9 ± 11.9	47.5 ± 9.87	23.5 ± 3.07 ^i^
*V*_ss_ (mL/kg)	126 ± 11.1	102 ± 8.16 ^e^	1130 ± 290	1290 ± 327	1100 ± 429	1760 ± 595	1600 ± 333	1470 ± 254
MRT (min)	28.2 ± 4.33	40.3 ± 4.73 ^e^	38.5 ± 8.43	53.5 ± 14.0	51.4 ± 18.9	32.1 ± 5.46	39.4 ± 15.2	63.1 ± 12.1 ^i^
Terminal half-life (min)	93.9 ± 18.7	91.5 ± 16.0	81.5 ± 17.4	157 ± 53.7	214 ± 111	77.8 ± 5.58	75.1 ± 18.2	77.7 ± 36.0
Ae_0–24h_ (% of dose)	6.94 ± 3.67	2.22 ± 1.16 ^d^	2.06 ± 1.87	0.941 ± 0.362	2.55 ± 1.78	0.0750 ± 0.0191	0.0986 ± 0.0785	0.0605 ± 0.0223
GI_24h_ (% of dose)	0.701 ± 1.17	0.481 ± 0.219	0.182 ± 0.124	0.186 ± 0.223	0.153 ± 0.165	0.0105 ± 0.00709	0.0103 ±0.00265	0.0153 ± 0.0106

^a^ Dose-normalized values were compared when statistical analysis was performed. ^b^ Dose-normalized value was significantly different from 2.3 mg/kg group (*p* < 0.01, *t*-test). ^c^ The value was significantly different from 2.3 mg/kg group (*p* < 0.001, *t*-test). ^d^ The value was significantly different from 2.3 mg/kg group (*p* < 0.05, *t*-test). ^e^ The value was significantly different from 2.3 mg/kg group (*p* < 0.01, *t*-test). ^f^ Dose-normalized value was significantly different from 14.8 mg/kg group (*p* < 0.05, one-way analysis of variance (ANOVA) with post hoc Tukey test). ^g^ The value was significantly different from 14.8 mg/kg group (*p* < 0.05, one-way ANOVA with post hoc Tukey test). ^h^ Dose-normalized value was significantly different from 2.9 and 5.8 mg/kg groups (*p* < 0.05, one-way ANOVA with post hoc Tukey test). ^i^ The value was significantly different from 2.9 and 5.8 mg/kg groups (*p* < 0.05, one-way ANOVA with post hoc Tukey test).

**Table 2:** Pharmacokinetic parameters of SSa after oral administration of pure SSa or DA-9805 at doses equivalent to 1.1, 2.3, and 4.6 mg/kg of SSa to rats (mean ± SD).

Parameters	Oral Doses				
	1.1 mg/kg as SSa	2.3 mg/kg as SSa		4.6 mg/kg as SSa	
	DA-9805 0.25 g/kg (*n* = 5)	Pure SSa 2.3 mg/kg (*n* = 4)	DA-9805 0.5 g/kg (*n* = 5)	Pure SSa 4.6 mg/kg (*n* = 5)	DA-9805 1 g/kg (*n* = 5)
Body weight (g)	257 ± 5.70	249 ± 6.29	252 ± 6.71	204 ± 9.62	212 ± 5.70
AUC_0–8h_ (μg∙min/mL) ^a^	0.285 ± 0.0948	0.206 ± 0.129	0.404 ± 0.0839 *	0.460 ± 0.0451	1.21 ± 0.586 *
AUC_0–10h_ (μg∙min/mL) ^a^				0.490 ± 0.0521	1.49 ± 0.769 *
*C*_max_ (μg/mL) ^a^	1.61 ± 0.248	3.00 ± 2.22	1.91 ± 0.808	4.67 ± 0.883	6.70 ± 3.46
*T*_max_ (min) ^b^	45 (15–60)	17.5 (5–30)	60 (60–120)	30 (15–45)	45 (15–480)
GI_24h_ (% of dose)	2.19 ± 5.47	1.99 ± 1.49	26.5 ± 6.99 ***	14.2 ± 12.4	44.4 ± 7.17 ***
F (%)		0.0399	0.0783	0.0251	0.0659

^a^ Dose-normalized values were compared when statistical analysis was performed. ^b^ *T*_max_ is expressed as median (range). * The value was significantly different from each same equivalent dose of pure SSa groups (* *p* < 0.05, *** *p* < 0.001; *t*-test).

**Table 3:** Pharmacokinetic parameters of PA after oral administration of pure PA or DA-9805 at doses equivalent to 14.8, 29.6, and 59.2 mg/kg of PA to rats (mean ± SD).

Parameters	Oral Doses					
	14.8 mg/kg as PA		29.6 mg/kg as PA		59.2 mg/kg as PA	
	Pure PA 14.8 mg/kg (*n* = 4)	DA-9805 0.25 g/kg (*n* = 5)	Pure PA 29.6 mg/kg (*n* = 5)	DA-9805 0.5 g/kg (*n* = 5)	Pure PA 59.2 mg/kg (*n* = 4)	DA-9805 1 g/kg (*n* = 5)
Body weight (g)	251 ± 12.5	257 ± 5.70	247 ± 6.71	252 ± 6.71	204 ± 7.50	212 ± 5.70
AUC_0–inf_ (μg∙min/mL) ^a^	82.4 ± 11.5	147 ± 129	153 ± 101	404 ± 83.9 *	462 ± 142	1180 ± 341 **
Terminal half-life (min)	79.1 ± 51.7	98.8 ± 56.1	70.5 ± 29.7	66.8 ± 15.6	50.5 ± 13.4	84.2 ± 55.4
*C*_max_ (μg/mL) ^a^	2.05 ± 0.956	1.05 ± 0.855	2.78 ± 1.46	2.48 ± 0.853	8.20 ± 2.63	4.17 ± 1.53 *
*T*_max_ (min) ^b^	15 (15)	30 (15–120)	15 (5–30)	60 (60–180)	5 (5–15)	120 (30–120)
GI_24h_ (% of dose)	0.406 ± 0.212	0.511 ± 1.01	0.0572 ± 0.0426	1.24 ± 1.39	0.119 ± 0.143	0.167 ± 0.128
F (%)	16.1	28.7	12.2	40.5	16.4	41.7

^a^ Dose-normalized values were compared when statistical analysis was performed. ^b^ *T*_max_ is expressed as median (range). * The value was significantly different from each same equivalent dose of pure PA groups (* *p* < 0.05, ** *p* < 0.01; *t*-test).

**Table 4:** Pharmacokinetic parameters of IMP after oral administration of pure IMP or DA-9805 at doses equivalent to 1.4, 2.9, and 5.8 mg/kg of DA-9805 to rats (mean ± SD).

Parameters	Oral Doses					
	1.4 mg/kg as IMP		2.9 mg/kg as IMP		5.8 mg/kg as IMP	
	Pure IMP 1.4 mg/kg (*n* = 6)	DA-9805 0.25 g/kg (*n* = 5)	Pure IMP 2.9 mg/kg (*n* = 6)	DA-9805 0.5 g/kg (*n* = 5)	Pure IMP 5.8 mg/kg (*n* = 5)	DA-9805 1 g/kg (*n* = 5)
Body weight (g)	258 ± 10.8	257 ± 5.70	253 ± 6.12	252 ± 6.71	204 ± 11.9	212 ± 5.70
AUC_0–inf_ (μg∙min/mL) ^a^	1.53 ± 0.851	3.02 ± 2.38	2.95 ± 3.13	6.74 ± 5.29	36.2 ± 18.1 ^#^	22.5 ± 12.6
Terminal half-life (min)	121 ± 38.3	120 ± 51.1	99.9 ± 31.5	73.8 ± 24.7	101 ± 29.2	56.5 ± 7.26 *
C_max_ (μg/mL) ^a^	17.7 ± 6.79	28.4 ± 20.8	58.6 ± 76.4	33.5 ± 25.7	568 ± 312 ^#^	88.2 ± 46.2 **
T_max_ (min) ^b^	22.5 (5–45)	60 (30–120)	30 (15–30)	240 (90–240)	30 (15–60)	240 (30–360)
GI_24h_ (% of dose)	0.0285 ± 0.0152	2.19 ± 5.47	0.153 ± 0.228	0.846 ± 0.854	1.37 ± 1.09	6.30 ± 2.89
F (%)			5.27	12.1	28.7	17.8

^a^ Dose-normalized values were compared when statistical analysis was performed. ^b^ *T*_max_ is expressed as median (range). ^#^ The dose-normalized value was significantly different from lower dose groups (^#^ *p* < 0.05; Kruskal-Wallis test). * The value was significantly different from each same equivalent dose of pure IMP group (*p < 0.05, ** *p* < 0.01; *t*-test).

## Abbreviations

Ae_0–24h_	the percentage of the dose excreted in the urine during 24 h
AUC	area under the plasma concentration–time curve
CL	time-averaged total body clearance
CL_NR_	time-averaged nonrenal clearance
CL_R_	time-averaged renal clearance
*C*_max_	maximum plasma concentration
F	the extent of absolute oral bioavailability
*f*_u_	unbound fraction
GI_24 h_	the percentage of the dose recovered from the gastrointestinal tract at 24 h (including their contents and feces during 24 h)
IMP	imperatorin
IS	internal standard
LC-MS/MS	liquid chromatographic–tandem mass spectrometry
LLOQ	lower limit of quantification
MRT	mean residence time
PA	paeonol
PKs	pharmacokinetics
SD	standard deviation
SSa	saikosaponin a
*T*_max_	time to reach *C*_max_
*V*_ss_	apparent volume of distribution at steady state

## References

[1] Amro M.S., Teoh S.L., Norzana A.G., Srijit D. (2018). The potential role of herbal products in the treatment of Parkinson’s disease. Clin. Ter..

[2] Francardo V., Schmitz Y., Sulzer D., Cenci M.A. (2017). Neuroprotection and neurorestoration as experimental therapeutics for Parkinson’s disease. Exp. Neurol..

[3] Park W.H., Kang S., Piao Y., Pak C.J., Oh M.S., Kim J., Kang M.S., Pak Y.K. (2015). Ethanol extract of *Bupleurum*
*falcatum* and saikosaponins inhibit neuroinflammation via inhibition of NF-κB. J. Ethnopharmacol..

[4] Lee J.Y., Kim H.S., Oh T.H., Yune T.Y. (2010). Ethanol extract of *Bupleurum*
*falcatum* improves functional recovery by inhibiting matrix metalloproteinases-2 and -9 activation and inflammation after spinal cord injury. Exp. Neurobiol..

[5] Lee B., Shim I., Lee H., Hahm D.H. (2009). Effect of *Bupleurum*
*falcatum* on the stress-induced impairment of spatial working memory in rats. Biol. Pharm. Bull..

[6] Kim H.G., Park G., Piao Y., Kang M.S., Pak Y.K., Hong S.P., Oh M.S. (2014). Effects of the root bark of *Paeonia*
*suffruticosa* on mitochondria-mediated neuroprotection in an MPTP-induced model of Parkinson’s disease. Food Chem. Toxicol..

[7] Moon Y.J., Lee J.Y., Oh M.S., Pak Y.K., Park K.S., Oh T.H., Yune T.Y. (2012). Inhibition of inflammation and oxidative stress by *Angelica*
*dahuricae* radix extract decreases apoptotic cell death and improves functional recovery after spinal cord injury. J. Neurosci. Res..

[8] Tang Y.H., Zhang Y.Y., Zhu H.Y., Huang C.G. (2007). A high-performance liquid chromatographic method for saikosaponin a quantification in rat plasma. Biomed. Chromatogr..

[9] Chen M.F., Huang C.C., Liu P.S., Chen C.H., Shiu L.Y. (2013). Saikosaponin a and saikosaponin d inhibit proliferation and migratory activity of rat HSC-T6 cells. J. Med. Food.

[10] Ye M., Bi Y.F., Ding L., Zhu W.W., Gao W. (2016). Saikosaponin a functions as anti-epileptic effect in pentylenetetrazol induced rats through inhibiting mTOR signaling pathway. Biomed. Pharmacother..

[11] Mao X., Miao G., Tao X., Hao S., Zhang H., Li H., Hou Z., Tian R., Lu T., Ma J. (2016). Saikosaponin a protects TBI rats after controlled cortical impact and the underlying mechanism. Am. J. Transl. Res..

[12] Liu Y., Li Z., Liu X., Pan R. (2014). Review on the toxic effects of Radix Bupleuri. Curr. Opin. Complement. Alternat. Med..

[13] Xu L., Song R., Tian J.X., Tian Y., Liu G.Q., Zhang Z.J. (2012). Analysis of saikosaponins in rat plasma by anionic adducts-based liquid chromatography tandem mass spectrometry method. Biomed. Chromatogr..

[14] Li H., Wang S., Yang Q., Xie Y., Cao W., Zhang B., Wang J., Wang J., Wang M. (2011). LC tissue distribution study of paeonol in rats after oral administration. Chromatographia.

[15] Li Y.J., Bao J.X., Xu J.W., Murad F., Bian K. (2010). Vascular dilation by paeonol—A mechanism study. Vasc. Pharmacol..

[16] Lee B., Shin Y.W., Bae E.A., Han S.J., Kim J.S., Kang S.S., Kim D.H. (2008). Antiallergic effect of the root of *Paeonia*
*lactiflora* and its constituents paeoniflorin and paeonol. Arch. Pharm. Res..

[17] Tsai H.Y., Lin H.Y., Fong Y.C., Wu J.B., Chen Y.F., Tsuzuki M., Tang C.H. (2008). Paeonol inhibits RANKL-induced osteoclastogenesis by inhibiting ERK, p38 and NF-kappaB pathway. Eur. J. Pharmacol..

[18] Hsieh C.L., Cheng C.Y., Tsai T.H., Lin I.H., Liu C.H., Chiang S.Y., Lin J.G., Lao C.J., Tang N.Y. (2006). Paeonol reduced cerebral infarction involving the superoxide anion and microglia activation in ischemia-reperfusion injured rats. J. Ethnopharmacol..

[19] Wu J.B., Song N.N., Wei X.B., Guan H.S., Zhang X.M. (2008). Protective effects of paeonol on cultured rat hippocampal neurons against oxygen-glucose deprivation-induced injury. J. Neurol. Sci..

[20] Zhong S.Z., Ge Q.H., Qu R., Li Q., Ma S.P. (2009). Paeonol attenuates neurotoxicity and ameliorates cognitive impairment induced by D-galactose in ICR mice. J. Neurol. Sci..

[21] Tsai T.H., Chou C.J., Chen C.F. (1994). Pharmacokinetics of paeonol after intravenous administration in rats. J. Pharm. Sci..

[22] Xie Y., Zhou H., Wong Y.F., Xu H.X., Jiang Z.H., Liu L. (2008). Study on the pharmacokinetics and metabolism of paeonol in rats treated with pure paeonol and an herbal preparation containing paeonol by using HPLC-DAD-MS method. J. Pharm. Biomed. Anal..

[23] Xie Y., Jiang Z.H., Zhou H., Ma W.Z., Wong Y.F., Liu Z.Q., Liu L. (2014). The pharmacokinetic study of sinomenine, paeoniflorin and paeonol in rats after oral administration of a herbal product Qingfu Guanjiesu capsule by HPLC. Biomed. Chromatogr..

[24] Xiao Y., Zhang Y.H., Sheng Y.X., Zhang J.L. (2008). LC-MS determination and pharmacokinetic studies of paeonol in rat plasma after administration of different compatibility of Su-Xiao-Xin-Tong prescriptions. Biomed. Chromatogr..

[25] Abad M.J., de las Heras B., Silvan A.M., Pascual R., Bermejo P., Rodriquez B., Villar A.M. (2001). Effects of furocoumarins from *Cachrys*
*trifida* on some macrophage functions. J. Pharm. Pharmacol..

[26] Ban H.S., Lim S.S., Suzuki K., Jung S.H., Lee S., Lee Y.S., Shin K.H., Ohuchi K. (2003). Inhibitory effects of furanocoumarins isolated from the roots of *Angelica*
*dahurica* on prostaglandin E2 production. Planta Med..

[27] Wang N., Wu L., Cao Y., Wang Y., Zhang Y. (2013). The protective activity of imperatorin in cultured neural cells exposed to hypoxia re-oxygenation injury via anti-apoptosis. Fitoterapia.

[28] Budzynska B., Boguszewska-Czubara A., Kruk-Slomka M., Skalicka-Wozniak K., Michalak A., Musik I., Biala G., Glowniak K. (2013). Effects of imperatorin on nicotine-induced anxiety- and memory-related responses and oxidative stress in mice. Physiol. Behav..

[29] Luszczki J.J., Glowniak K., Czuczwar S.J. (2007). Time-course and dose-response relationships of imperatorin in the mouse maximal electroshock seizure threshold model. Neurosci. Res..

[30] Zhao G., Peng C., Du W., Wang S. (2014). Simultaneous determination of imperatorin and its metabolites in vitro and in vivo by a GC-MS method: Application to a bioavailability and protein binding ability study in rat plasma. Biomed. Chromatogr..

[31] Zhang J., Zhang M., Fu S., Li T., Wang S., Zhao M., Ding W., Wang C., Wang Q. (2014). Simultaneous determination of imperatorin and its metabolite xanthotoxol in rat plasma by using HPLC-ESI-MS coupled with hollow fiber liquid phase microextraction. J. Chromatogr. B Anal. Technol. Biomed. Life Sci..

[32] Zhang X., Xie Y., Cao W., Yang Q., Miao S., Wang S. (2011). Brain distribution study of imperatorin in rats after oral administration assessed by HPLC. Chromatographia.

[33] Lili W., Yehong S., Qi Y., Yan H., Jinhui Z., Yan L., Cheng G. (2013). In vitro permeability analysis, pharmacokinetic and brain distribution study in mice of imperatorin, isoimperatorin and cnidilin in Radix *Angelicae dahuricae*. Fitoterapia.

[34] Li Y., Wang Y., Tai W., Yang L., Chen Y., Chen C., Liu C. (2015). Challenges and solutions of pharmacokinetics for efficacy and safety of traditional Chinese medicine. Curr. Drug Metab..

[35] Kim J.M., Yoon J.N., Jung J.W., Choi H.D., Shin Y.J., Han C.K., Lee H.S., Kang H.E. (2013). Pharmacokinetics of hederacoside C, an active ingredient in AG NPP709, in rats. Xenobiotica.

[36] Kwon M.H., Jeong J.S., Ryu J., Cho Y.W., Kang H.E. (2017). Simultaneous determination of saikosaponin a, paeonol, and imperatorin, components of DA-9805, in rat plasma by LC-MS/MS and application to a pharmacokinetic study. J. Chromatogr. B Anal. Technol. Biomed. Life Sci..

[37] Chiou W.L. (1978). Critical Evaluation of the potential error in pharmacokinetic studies of using the linear trapezoidal rule method for the calculation of the area under the plasma level–time curve. J. Pharmacokinet. Biopharm..

[38] Gibaldi M., Perrier D. (1982). Pharmacokinetics.

[39] Davies B., Morris T. (1993). Physiological parameters in laboratory animals and humans. Pharm. Res..

[40] Shimizu K., Amagaya S., Ogihara Y. (1985). Structural transformation of saikosaponins by gastric juice and intestinal flora. J. Pharmacobiodyn..

[41] Kida H., Akao T., Meselhy M.R., Hattori M. (1998). Metabolism and pharmacokinetics of orally administered saikosaponin b1 in conventional, germ-free and *Eubacterium* Sp. A-44-infected gnotobiote rats. Biol. Pharm. Bull..

[42] Cao Y., Zhong Y.H., Yuan M., Li H., Zhao C.J. (2013). Inhibitory Effect of imperatorin and isoimperatorin on activity of cytochrome p450 enzyme in human and rat liver microsomes. Zhongguo Zhong Yao Za Zhi.

[43] Liu H.X., Hu Y., Liu Y., He Y.Q., Li W., Yang L. (2009). CYP1A2 is the major isoform responsible for paeonol *O*-demethylation in human liver microsomes. Xenobiotica.

[44] Liu G., Tian Y., Li G., Xu L., Song R., Zhang Z. (2013). Metabolism of saikosaponin a in rats: Diverse oxidations on the aglycone moiety in liver and intestine in addition to hydrolysis of glycosidic bonds. Drug Metab. Dispos..

[45] Chen L.C., Chou M.H., Lin M.F., Yang L.L. (2001). Effects of Paeoniae Radix, a traditional Chinese medicine, on the pharmacokinetics of phenytoin. J. Clin. Pharm. Ther..

[46] Kobayashi M., Ueda C., Aoki S., Tajima K., Tanaka N., Yamahara J. (1990). Anticholinergic action of paeony root and its active constituents. Yakugaku Zasshi.

[47] Jung B.S., Shin M.K. (2003). Korean Domestic Pharmaceutical Pharmacopoeia (Herbal Medicine).

